# ﻿*Hemilophiacardiocarpa* (Brassicaceae), a new species from Yunnan, southwest China

**DOI:** 10.3897/phytokeys.194.82737

**Published:** 2022-04-18

**Authors:** Shao-Yun Liu, Zheng-Tao Ren, Chang-You Zhao, Chun-Xiang Hu, Huan-Chong Wang

**Affiliations:** 1 School of Ecology and Environmental Science, Yunnan University, Kunming 650500, Yunnan, China Yunnan University Kunming China; 2 Management Bureau of Yunnan Jiaozishan National Natural Reserve, Luquan, 651515, Yunnan, China Management Bureau of Yunnan Jiaozishan National Natural Reserve Luquan China; 3 Herbarium of Yunnan University, Kunming 650091, Yunnan, China Herbarium of Yunnan University Kunming China

**Keywords:** Alpine flora, Cruciferae, endemism, *Hemilophiarockii*, Jiaozishan Mountains

## Abstract

*Hemilophiacardiocarpa* (Brassicaceae), the sixth species of the Chinese endemic genus *Hemilophia*, is described and illustrated. This plant is found in the Jiaozishan Mountains in Dongchuan District, northern Yunnan, southwest China. Morphologically, it shows close relationships with *H.rockii* and *H.pulchella*, but differs from it in the leaf shape and size, inflorescence, flower size, shape of fruit and length of its pedicel. An updated key to the taxa of *Hemilophia* is provided.

## ﻿Introduction

*Hemilophia* Franch. is a small genus established by the French botanist Adrien René Franchet based on Delavay’s specimens from Lijiang, southwest China (Franchet 1889). Currently, five species of this genus are recognized, namely *H.franchetii* Al-Shehbaz, *H.pulchella* Franch., *H.rockii* O. E. Schulz, *H.serpens* (O. E. Schulz) Al-Shehbaz, *H.sessilifolia* Al-Shehbaz, Kats. Arai & H. Ohba (Zhou et al. 2001). All are restricted to high-elevation habitats of the Hengduan Mountains in southwest China (Zhou et al. 2001; Al-Shehbaz 2015). The systematic position of *Hemilophia* in Brassicaceae is not well resolved up to now. Schulz (1936) placed it in the tribe Lepidieae DC., whereas this treatment was not supported by recent studies (Al-Shehbaz 2012; Nikolov et al. 2019; Walden et al. 2020; Francis et al. 2021; Liu et al. 2021).

During our botanical fieldwork to Jiaozishan Mountains in southwest China in August 2018, a showy plant of *Hemilophia* with cordiform fruits was collected. By thoroughly examining the specimens of this genus housed at herbaria KUN, PE and YUKU, and a survey of digital images available at the database Plant Photo Bank of China (http://ppbc.iplant.cn/), we found that this plant had been collected or photographed several times in Jiaozishan Mountains and was wrongly identified as *H.rockii* O. E. Schulz. Comparison with related species demonstrates that this plant actually represents a distinct species hitherto not described.

## ﻿Materials and methods

The study followed the normal practice of plant taxonomic survey and herbarium taxonomy. Morphological studies of the new species were based on observation of living plants and specimens from the Jiaozishan Mountains in Dongchuan District, northern Yunnan, southwest China. Digital images of type specimens of genus *Hemilophia* available at the JSTOR Global Plants (http://plants.jstor.org/), as well collections housed at CDBI, KUN, PE, PYU and YUKU were examined and compared with the new species. Pertinent taxonomic literature (e.g., Al-Shehbaz 1999, 2002, 2015; Zhou et al. 2001) was extensively consulted. Measurements were carried out under a stereomicroscope (Olympus SZX2, Tokyo, Japan) using a ruler and a metric vernier caliper.

## ﻿Taxonomy

### ﻿ Hemilophia cardiocarpa

Taxon classificationPlantaeBrassicalesBrassicaceae

Huan C. Wang, Shao Y. Liu & Z. T. Ren sp. nov.

9DC98602-6992-57AD-8125-9D11B667F0A2

urn:lsid:ipni.org:names:77297069-1

Figs 1 , 2 , 3

#### Type.

China. Yunnan Province: Dongchuan District, Jiaozishan Mountains, on screes, 26°9'45"N, 102°56'7"E, alt. 3,970–4,000 m, 7 September 2018, *Huan-Chong Wang et al. LQ4146* (Holotype: YUKU!; Isotypes: YUKU!)

#### Diagnosis.

*Hemilophiacardiocarpa* is most similar to *H.rockii*, but clearly differs from the latter by its cauline leaves 5–8 mm long (vs. 3–6 mm long in *H.rockii*), 2–4 mm (vs. 1–3 mm) wide, pedicel of fruit shorter than or nearly equal to its leaflike bract (vs. significantly longer than leaflike bract) and fruit cordate (vs. spindle or narrowly oblong) in shape.

#### Description.

Perennial herbs, cespitose, rhizomatous. Rhizomes slender, simple or branched, glabrous. Stems simple or few branched, 3–10 cm in length, with appressed, simple and minutely forked, 0.05–0.2 mm long trichomes. Basal leaves rosulate, obovate to oblanceolate, withering and deciduous at anthesis. Cauline leaves alternate; petioles blue-purple, glabrous, 1–2.5 mm long; blade ovate, obovate or oblong, 5–8 mm long, 2–4 mm wide, base cuneate, margin entire, apex usually acute to broadly acute, rarely obtuse; midveins sparsely pubescent adaxially, lateral nerves indistinct. Racemes terminal, usually 5–13-flowered. Pedicels 1–3 mm long, with dense and minute hairs. Sepals oblong or ovate, ascending, equal, caducous in fruit, 1–2 mm long, 0.8–1.25 mm wide, sparsely pubescent abaxially, margins membranous and ciliate, rounded at apex. Petals alternate with sepals, broadly obovate, bluish-white to white, with blue or a few purple veins on the lower half, 4–7 mm long, 2–3.5 mm wide, abruptly narrowed to claw at base, apex shallowly to deeply emarginate, apical notch up to 0.4–0.5 mm in depth. Stamens 6, in 2 whorls, slightly tetradynamous; filaments light yellow to purple, lateral pair slender, 1.2–1.5 mm long, median pairs 1.1–1.5 mm long, strongly dilated and appendaged at base; anthers nearly dark purple, 0.45–0.5 mm long, longitudinally dehiscent. Nectar glands surrounding base of lateral stamens, subtending base of median filaments. Pistil 2-carpelled, ovary sessile, ovate; style cylindric, minutely papillate, nearly equal length to stamens. Pedicel of fruit elongated in fruit stage, 4–6 mm long, shorter than or nearly equal to leaflike bract in length. Fruit cordate in shape, glabrous, dehiscent, 5–5.5 mm long, 3–3.5 mm wide; valves navicular, thinly papery, with a crest of tubercles surrounding margin and extending along midvein; replum slender, 5.3–5.5 mm long; septum absent. Seeds 2 per fruit, usually ovoid, slightly flattened, 3.4–3.8 mm long.

**Figure 1.**
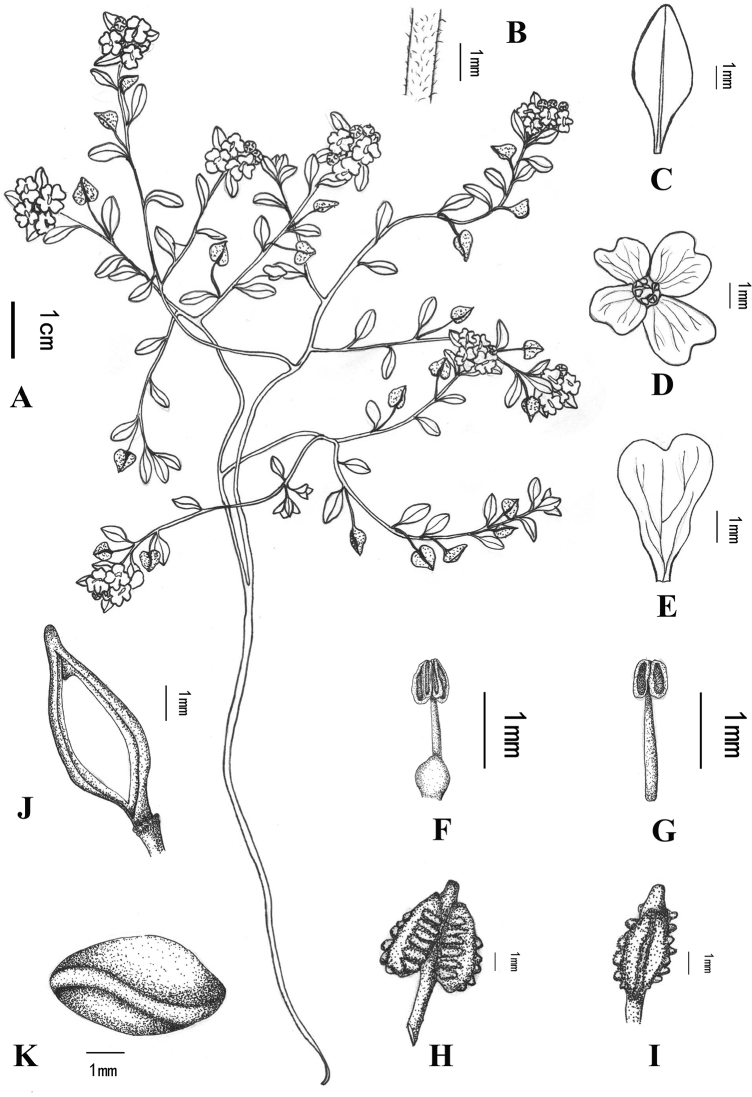
*Hemilophiacardiocarpa***A** habit **B** a portion of stem showing trichomes **C** cauline leaf **D** flower (apical view) **E** petal **F** median filament **G** lateral filament **H** fruit (front view) **I** fruit (lateral view) **J** fruit after removal of valves and seeds **K** seed.

#### Phenology.

Flowering occurs from May to early September, and fruiting from August to October.

#### Etymology.

The specific epithet *cardiocarpa* is derived from the Greek words “*kardio*” (heart) and “*karpos*” (fruit), referring to the fruit shape of this new species.

**Figure 2.**
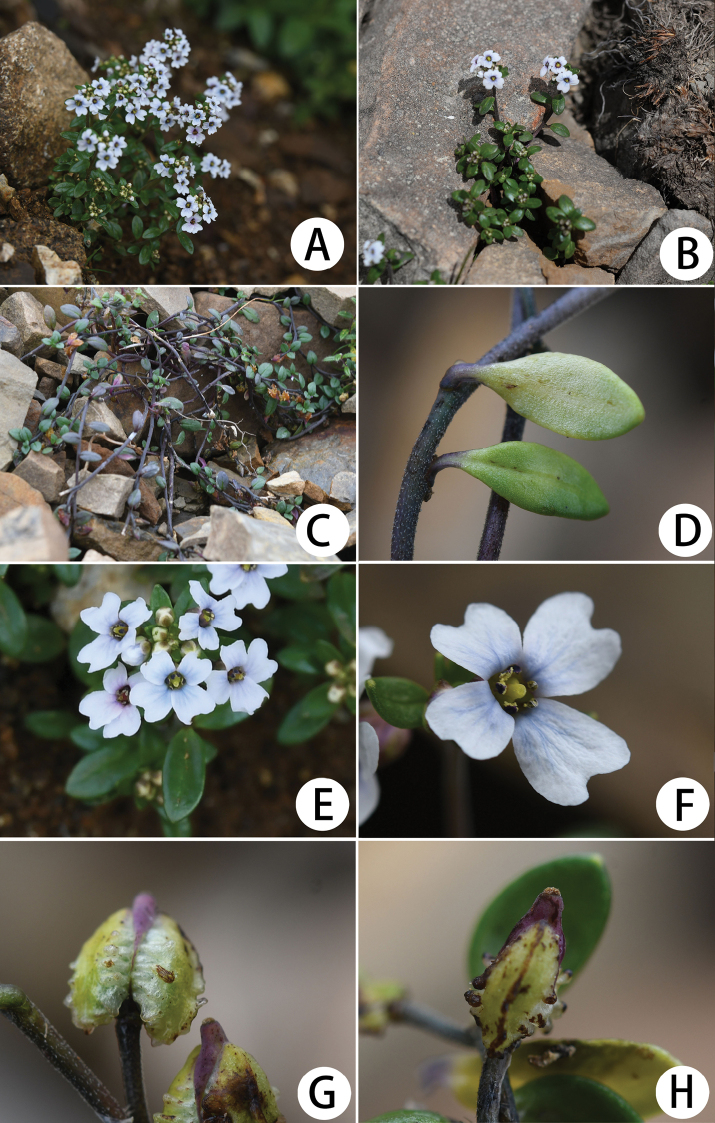
*Hemilophiacardiocarpa***A–C** habit **D** a portion of stem with two cauline leaves **E** inflorescences in apical view **F** flower **G** fruit (front view) **H** fruit (lateral view).

#### Vernacular name.

Chinese Mandarin: xin guo ban ji ji (心果半脊荠).

#### Distribution and ecology.

*Hemilophiacardiocarpa* appears to be a rare species endemic to the Jiaozishan Mountains, which are located in the northern Yunnan Province of southwest China with a highest elevation of 4344.1m and near to the Jinsha River. The new species grows on basaltic screes or open slopes at elevations ranging from 3900 to 4300 m, its association mainly include *Arenariaweissiana* Hand-Mazz. (Caryophyllaceae), *Drabaamplexicaulis* Franch. (brassicaceaee), *Scrophulariadelavayi* Franch.(Scrophulariaceae), *Meconopsisintegrifolia* (Maxim.) Franch. (Papaveraceae), Ranunculushirtellusvar.orientalis W. T. Wang (Ranunculaceae) and Veronicaszechuanicasubsp.sikkimensis (Hook.f.) Hong (Plantaginaceae).

**Figure 3.**
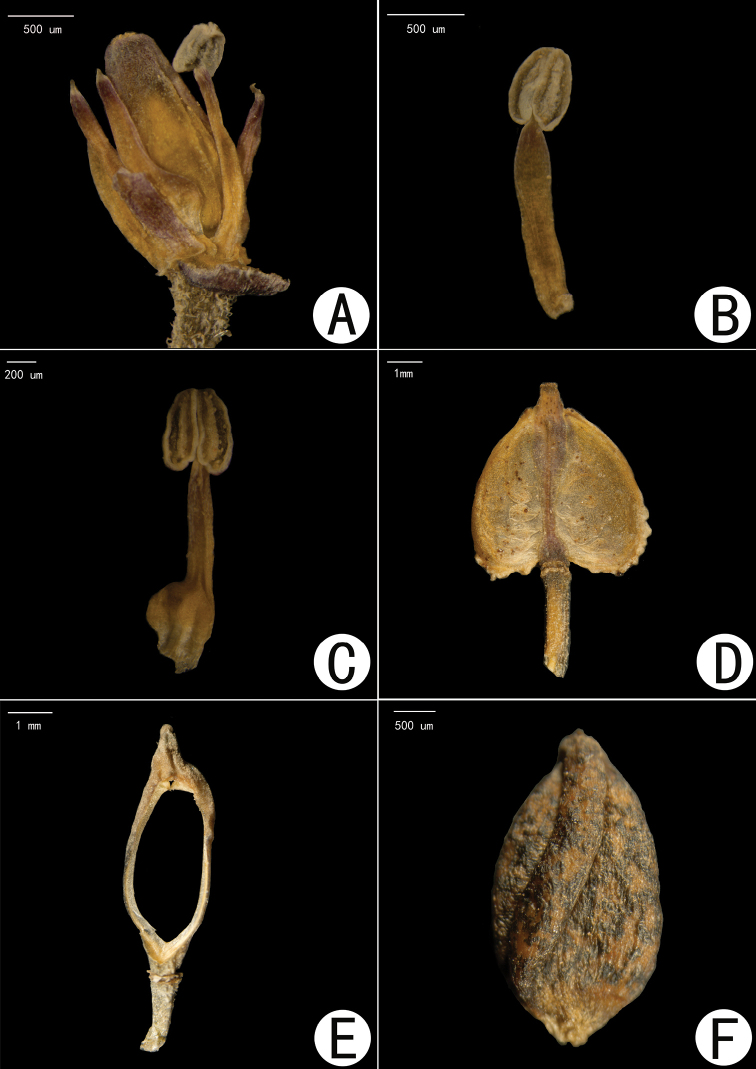
*Hemilophiacardiocarpa***A** flower after removal of sepals and petals **B** lateral filament **C** median filament **D** fruit (front view) **E** fruit after removal of valves and seeds **F** seed.

#### Additional specimens examined

**(paratypes)**: **China. Yunnan**: Dongchuan District, Jiaozishan Mountains, Lanniping, elev. 4300 m, 25 August. 1985, *Lan Shunbin 547* (PE); Dongchuan District, Jiaozishan Mountains,Yaojingtan, elev. 4100 m, 25 June. 2020, *H. C. Wang et al. DC8448* (YUKU); Dongchuan District, Jiaozishan Mountains, Jinfengkou, elev. 4200 m, 23 May 2021, *Ren Zhengtao et al. DC12360* (YUKU).

#### Taxonomic notes.

**Figure 4.**
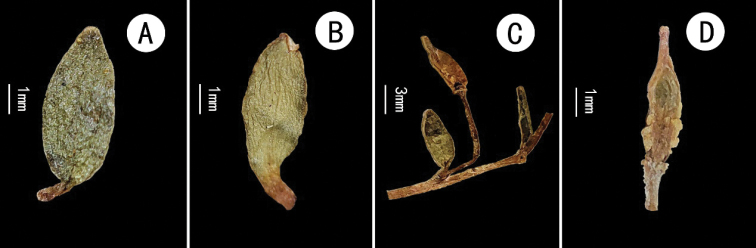
*Hemilophiarockii***A** acauline leaf showing adaxial face **B** cauline leaf showing abaxial face **C** a portion of infructescence **D** fruit.

The presence of cordiform fruits is the most remarkable character to distinguish *Hemilophiacardiocarpa* from other species of this genus. Morphologically, *H.cardiocarpa* is most similar to *H.rockii* (Fig. 4) in having a similar habit and sharing similar indumentum, leaf shape, as well as flower size and arrangement. Nevertheless, it clearly differs from the latter by its cauline leaves usually ovate, rarely obovate or oblong (vs. oblanceolate to narrowly elliptic, or rarely ovate in *H.rockii*), 5–8 mm (vs. 3–4 (6)) long, 2–4 mm (vs. 1–1.5 (3)) wide, racemes usually 5–13 (vs. 5–6) -flowered, pedicels of fruits shorter than or nearly equal to its leaflike bract (vs. significantly longer than leaflike bract) and fruit cordate (vs. spindle or narrowly oblong) in shape. *H.cardiocarpa* is also similar to the type species of the genus, *H.pulchella* Franch., from which it is readily distinguished by the racemes usually 5–13-flowered (vs. 2–3-flowered in *H.pulchella*), and petals bluish-white to white (vs. pink), 4–7 mm (vs. 2.5–3.5 mm) long, 2–3.5 mm (vs. 1.5–2 mm) wide. Taxa of *Hemilophia* can be distinguished through the morphological characters presented in the following identification key modified from Al-Shehbaz (2002, 2015).

### ﻿Identification key to the species of *Hemilophia*

**Table:** 

1a	Cauline leaves sessile; sepals not ciliate at margin; style conical, glabrous.	
2a	Stems pubescent with simple trichomes only; petals creamy white with dark green veins; median filaments strongly inflated basally into an oblong appendage; ovules 2 per ovary	***H.sessilifolia***
2b	Stems pubescent exclusively with appressed, subsessile, forked and trifid trichomes; petals bright yellow with veins of same color as rest of limb; median filaments unappendaged at base; ovules 4 per ovary	***H.serpens***
1b	Cauline leaves petiolate; sepals ciliate at margin, if not then petals purple; style cylindric, minutely papillate.	
3a	Petals purplish; leaves with setose, appressed trichomes; sepals not ciliate; stem trichomes malpighiaceous, not crisped	***H.franchetii***
3b	Petals pink, creamy white or yellowish; leaves glabrous or with crisped pilose trichomes; sepals ciliate; stem trichomes puberulent, crisped.	
4a	Petals 2.5–3.5 mm long, 1.5–2 mm wide, pink, narrowly obovate, shallowly emarginate; leaves glabrous or rarely sparsely pilose	***H.pulchella***
4b	Petals 4–7 mm long, 2–5 mm wide, creamy white to yellowish, obcordate, deeply emarginate to nearly 2-lobed; leaves pilose or rarely glabrescent.	
5a	Petals creamy white; cauline leaves 5–8 mm long, 2–4 mm wide; pedicels of fruits shorter than or nearly equal length to leaflike bracts; fruits cordate in shape	***H.cardiocarpa***
5b	Petals creamy white to yellowish; cauline leaves 3–4 (6) mm long, 1–1.5 (3) mm wide; pedicels of fruits significantly longer than leaflike bracts; fruits spindle or narrowly oblong in shape	***H.rockii***

## Supplementary Material

XML Treatment for Hemilophia cardiocarpa
